# Pre-Acclimation to Elevated Temperature Stabilizes the Activity of Photosystem I in Wheat Plants Exposed to an Episode of Severe Heat Stress

**DOI:** 10.3390/plants11050616

**Published:** 2022-02-24

**Authors:** Andrej Filaček, Marek Živčák, Lorenzo Ferroni, Mária Barboričová, Kristína Gašparovič, Xinghong Yang, Marco Landi, Marián Brestič

**Affiliations:** 1 Department of Plant Physiology, Faculty of Agrobiology and Food Resources, Slovak University of Agriculture, Trieda A. Hlinku 2, 949 76 Nitra, Slovakia; xfilacek@uniag.sk (A.F.); xbarboricova@uniag.sk (M.B.); kristina.gasparovic@uniag.sk (K.G.); marian.brestic@uniag.sk (M.B.); 2 Laboratory of Plant Cytophysiology, Department of Environmental and Prevention Sciences, University of Ferrara, Corso Ercole I d’Este 32, 44100 Ferrara, Italy; 3 State Key Laboratory of Crop Biology, College of Life Science, Shandong Agricultural University, Taian 271018, China; xhyang@sdau.edu.cn; 4 Department of Agriculture, Food and Environment, University of Pisa, 56124 Pisa, Italy; marco.landi@agr.unipi.it; 5 Department of Botany and Plant Physiology, Faculty of Agrobiology, Food and Natural Resources, Czech University of Life Sciences Prague, Kamycka 129, 165 00 Prague, Czech Republic

**Keywords:** photosynthesis, heat stress, acclimation, chlorophyll fluorescence, wheat

## Abstract

The importance of high temperature as an environmental factor is growing in proportion to deepening global climate change. The study aims to evaluate the effects of long-term acclimation of plants to elevated temperature on the tolerance of their photosynthetic apparatus to heat stress. Three wheat (*Triticum* sp. L.) genotypes differing in leaf and photosynthetic traits were analyzed: Thesee, Roter Samtiger Kolbenweizen, and ANK 32A. The pot experiment was established in natural conditions outdoors (non-acclimated variant), from which a part of the plants was placed in foil tunnel with elevated temperature for 14 days (high temperature-acclimated variant). A severe heat stress screening experiment was induced by an exposition of the plans in a growth chamber with artificial light and air temperature up to 45 °C for ~12 h before the measurements. The measurements of leaf photosynthetic CO_2_ assimilation, stomatal conductance, and rapid kinetics of chlorophyll a fluorescence was performed. The results confirmed that a high temperature drastically reduced the photosynthetic assimilation rate caused by the non-stomatal (biochemical) limitation of photosynthetic processes. On the other hand, the chlorophyll fluorescence indicated only a moderate level of decrease of quantum efficiency of photosystem (PS) II (Fv/Fm parameter), indicating mostly reversible heat stress effects. The heat stress led to a decrease in the number of active PS II reaction centers (RC/ABS) and overall activity o PSII (PI_abs_) in all genotypes, whereas the PS I (parameter ψ_REo_) was negatively influenced by heat stress in the non-acclimated variant only. Our results showed that the genotypes differ in acclimation capacity to heat stress, and rapid noninvasive techniques may help screen the stress effects and identify more tolerant crop genotypes. The acclimation was demonstrated more at the PS I level, which may be associated with the upregulation of alternative photosynthetic electron transport pathways with clearly protective functions.

## 1. Introduction

Heat stress is one of the main abiotic stresses that negatively affects agricultural areas around the world due to the induction of a wide range of adverse physiological, biochemical, morphological anatomical, and genetic reactions of plants [1]. The global increase in average temperature is expected to occur in line with more intense and frequent heat waves [2]. The effect of high temperatures on photosynthesis significantly determines the effects of global warming on crop yields [3,4]. The reduction in crop productivity under high temperatures is mainly due to reduced photosynthetic carbon assimilation. In particular, a plant is considered under heat stress if exposed to temperatures above a certain limit for a period sufficiently long enough to induce irreversible changes [5]. Plants are exposed to a wide range of temperatures in natural conditions, whether during the day, between days, or seasons. This temperature variability directly affects the performance because the speed of all biochemical processes increases until the optimal temperature is reached [6]. The temperature optimum for wheat growth is between 17 and 23 °C [7], whereas heat stress occurs above a limit temperature between 31 and 35 °C [8]. High temperatures cause denaturation and aggregation of proteins and increase the fluidity of membrane lipids. Well-known consequences are comprised of inactivation and/or degradation of enzymes in chloroplasts, mitochondria and other cell compartments inhibition of protein synthesis, and loss of membrane integrity [9]. Along with the direct effect of high temperature, further injuries are due to the production of toxic compounds and reactive oxygen species, as well as to reduced ion fluxes and starvation symptoms, resulting in overall growth inhibition [5]. The collapse of the cell structures and the consequent cell death are outcomes of a severe heat stress [10].

Photosynthesis is extremely sensitive to heat and can even undergo a complete inhibition. Its high temperature tolerance is challenged by sufficiently long hot periods and/or temperatures large exceeding of the optimum. The irreversible or slowly reversible damage affecting the photosynthetic biochemistry and structures, particularly the thylakoid membrane integrity, is the consequence of acute, chronic, and fluctuating heat stress [11,12]. The reduction in photosynthesis is primarily due to the damage of photosystem II (PSII) activity, which is the most heat-sensitive component of the photosynthetic electron transport chain [13,14]. PSII damage may be targeted at the D1 protein or the oxygen-evolving complex [15]. In addition, the rate of repair of damaged PSII is adversely affected, and therefore the inactivated PSII complexed progressively accumulate in the leaves [16]. Direct damage and impaired repairing of PSII result in a diminished electron flow into the transport chain and insufficient supply of reducing power for the carbon fixing reactions (for review, [6]). However, there are significant differences in temperature resistance between species and genotypes, which may be related to PSII thermostability variability [17,18]. Differently, photosystem I (PSI) is relatively resistant to high temperatures [19]. In particular, cyclic electron transport around PSI was suggested to be involved in the thermostability of PSI, which might reduce the risk of electron accumulation in the electron transport chain and, accordingly, the chance for PSI irreversible damage [20].

An important role in the response to heat stress is played by gas exchange between the atmosphere and the mesophyll, in particular the stomata behavior. A proper regulation of stomatal opening upon heat stress should allow continuity of photosynthesis and avoid excess water loss. These two needs are hardly balanced, and this cooperates with heat stress sensitivity. On one hand, due to the increased temperature stomata tending to be close, the stomatal conductance (gs) decreases and preserves the water balance of the plants [21]. However, closed stomata affect the thermic regulation of the leaf tissues, which results in dangerously high leaf temperatures. Therefore, on the contrary, the value of gs can be increased by the action of high temperature, thus avoiding insufficient cooling and CO_2_ supply for photosynthesis, but exposing plants to excessive water loss [22]. In addition, even mild heat stress can reduce the activation state of the ribulose-1,5 bisphosphate carboxylase-oxygenase (RuBisCO), which needs an active photosynthetic electron flow [23]. Lower RuBisCO activity results in a decrease in the stomatal conductance and, at the same time, a loss of net photosynthesis in many plant species [24].

It is well-established that the plant resistance against heat stress depends on complex combinations of genetic factors and phenotypic thermal acclimation. The latter can be considered as the result of the specific “thermal history” of the plant. With prolonged temperature rise, most plants (including wheat) can indeed adjust or acclimate their photosynthetic properties [25]. Photosynthetic thermal acclimation probably involves altered modified activity of the enzymes responsible for the fixation of CO_2_-primarily Rubisco-, and modifications of the electron transfer through PSII in chloroplasts [26], and changes in PSII sensitivity to photoinhibition [27]. Not all genotypes within a species have the same ability to tolerate heat stress. There is significant variability between species and within species, which opens up opportunities to improve crops’ thermal stress tolerance in crop breeding [4,28]. However, this will require more information on the mechanisms involved, as well as on the traits and techniques efficient in the screening and phenotyping process. A major part of the past research has focused on static temperature responses, obtaining information from plants exposed to a single heat stress event, generally followed by a recovery to optimal temperatures [6]. Such approaches are surely useful and have built our current understanding of photosynthesis under heat stress. However, a more close-to-truth phenotypization of photosynthetic responses can be achieved taking into account the importance of the “thermal history” of genotypes. In particular, we hypothesize that an episode of acute and severe heat stress can be better tolerated by plants already acclimated to temperatures chronically, but not severely, above the optimum. An experimental setup considering such fluctuation should be better suited to the screening of heat-resistant wheat genotypes. In this respect, our study aimed at providing physiological evidence on the effects of the long-term temperature pre-acclimation on photosynthetic responses of wheat to a severe heat stress episode, specifically targeting the genotype-related responses and acclimation observed at the level of PSII photochemistry. To test this experimental design, we used three contrasting genotypes of *Triticum* sp., selected according to the results of our previous studies focused on drought [29] and recovery after heat stress [30]. Applying a different stress scenario, we obtained new information contributing to a better understanding of the heat stress tolerance of crop plants.

## 2. Material and Methods

### 2.1. Plant Material and Growth Conditions

Three genotypes of winter wheat (*Triticum* sp.): Thesee (*Triticum aestivum* L., Germany), Roter Samtiger Kolbenweizen (*Triticum compactum* Host., Germany) and ANK 32A (*Triticum aestivum* L., Russia) were used to study the thermotolerance of wheat genetic resources and to test the sensitivity of selected parameters. The genotypes were pre-selected from a larger tested collection of wheat genotypes, considering the divergence in stress response and capacity to recover after stress shown in previous seasons under different stress scenarios. The seeds were sown individually in pots with a peat substrate with neutral pH (Klassman-TS1) in autumn and vernalized in natural conditions outdoors. After winter, the plants with substrate were transferred into larger pots (3 L) with the same substrate and slowly releasing fertilizer Osmocote Plus 15-9-12 (The Scotts Company Ltd., Thorne, UK), in which the plants were grown outdoors, exposed to natural climatic conditions and full sunlight. The pots were arranged in a block and were regularly irrigated to eliminate dehydration. The phenotype of the plants of three genotypes in a growth stage, in which the measurements were started, is shown in Supplementary Figure S1. 

A high-temperature acclimation started as soon as all plants had fully developed flag leaves. Acclimation was induced by moving half of all plants into a polyethylene film foil tunnel with the high light transmission (>90% light transmission at noon). The temperature inside the tunnel was 3–8 °C above the ambient temperature. The maximum temperature in the acclimation period in open air was 30 °C, whereas the maximum temperature reached 38 °C in the tunnel. The plants were exposed to an elevated temperature for 14 days before the initial measurements were made in laboratory conditions. After that, the plants of both variants (nonacclimated—NA; temperature acclimated—TA) were transferred to a growth chamber with artificial actinic light (light tubes Silvania, PAR intensity 200 µmol m^−2^ s^−1^) and exposed for ~12 h to severe heat stress, raising the air temperature to 45 °C. Based on results of previous experiments, the air temperature of 45 °C applied in a growth chamber used in this study provided efficient threshold conditions to distinguish heat tolerant and susceptible genotypes of wheat [30]. After the treatment, the measurements of parameters were repeated. 

Both variants (NA, TA) were represented by ten pots of each genotype. All 3 L pots contained a single wheat plant with several tillers (see Supplementary Figure S1). One pot (plant) represented one replicate; the experiments and measurements were performed in 10 replicates (*n* = 10) per variant and genotype. Measurements realized in all tested plants before the heat treatment at 45 °C were denoted as C (control), and measurements in all tested plants during exposure at 45 °C were denoted as H (heat stress).

### 2.2. Applied Measurements

#### 2.2.1. Gas Exchange Measurements

The direct measurements of leaf photosynthesis were performed using an infra-red gas analyzer Licor-6400XT (Li-Cor, Lincoln, NE, USA) Analyses were carried out under conditions of ambient temperature fixed in the measuring chamber of the instrument (23 °C or 45 °C) and ambient humidity (~60%). CO_2_ concentration in the growth chamber was set to 400 µmol mol^−1,^ and the light intensity was 1200 µmol m^−2^ s^−^^1^. Parameters were measured at the steady-state. The measured parameters were:

- A, the CO_2_ assimilation rate (μmol CO_2_ m^−2^ s^−1^),
- g_s_, stomatal conductance (mol H_2_O m^−2^ s^−1^),
- A/Ci, photosynthetic rate per unit of internal CO_2_ concentration ratio.

#### 2.2.2. Measurements of PSII and PSI Activity

The structural and functional changes related to PSII photochemistry were analyzed using fast chlorophyll *a* fluorescence measurements recorded using a portable fluorimeter Handy PEA (Hansatech, King’s Lynn, Norfolk, UK). A 15-min dark acclimation period was allowed using the leaf clips of the instrument; at the end, a 1-s saturation pulse of 3500 μmol ^−2^ s^−1^ was applied. For each plant, the flag leaf was analyzed at two central positions of the blade, as technical replicates. The measured data were analyzed using the JIP test [31,32]. From the multiple biophysical parameters characterizing the test, the following parameters were used for the analyses [33]:

- Fv/Fm, maximum quantum yield of PSII photochemistry measured in dark-adapted state;
- RC/ABS, a number of active PSII reaction centers per absorbed light unit;
- ψ_REo_, the probability of electron transfer from PS II beyond the PS I;
- PI_tot_, performance index

Activity of PSI was measured with a Dual-PAM-100 system (Heinz Walz, Effeltrich Germany). All samples were dark-adapted for 10 min prior to measurements. A saturating pulse (10,000 μmol(photon) m^−2^ s^−1^, duration of 300 ms) was applied to detect the maximal change in P700 signal (Pm) after application of the saturating pulse after far-red preillumination for 10 s according to the methods of Klughammer and Schreiber [34]

### 2.3. Statistics

For the statistical treatment of data, analysis of variance (ANOVA), followed by the posthoc Tukey HSD test (*p* < 0.05), was performed using the Statistica version 9.0 software (Statsoft Inc., Tulsa, OK, USA). The factors analyzed were “acclimation” (acclimated vs. non-acclimated group), “heat stress” (before vs. after heat stress treatment), and “genotype” (Thesee, Roter Samtiger, ANK 32A). The data presented in graphs represent the mean values ± standard error. Ten individuals of each genotype were analyzed using the noninvasive methods described in the previous paragraphs.

## 3. Results and Discussion

**Figure 1:**
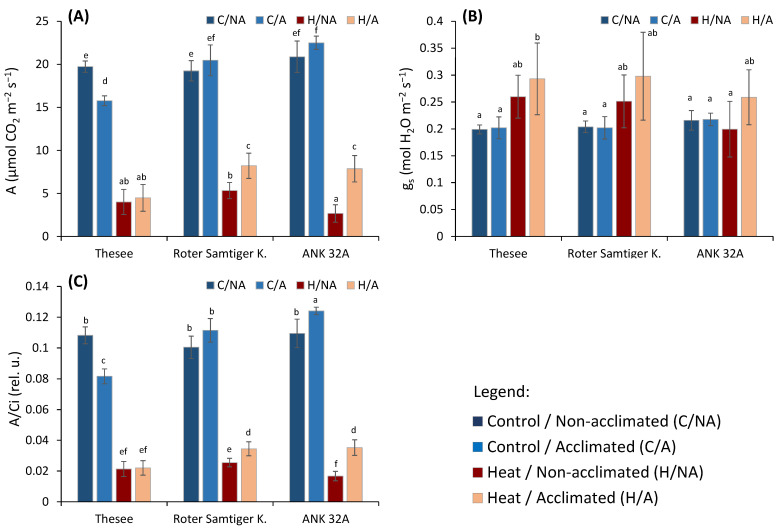
The effect of pre-acclimation and the heat stress treatment on selected photosynthetic parameters measured by infra-red gas analyzer: (**A**) CO_2_ assimilation rate (**A**); (**B**) stomatal conductance (gs); (**C**) A/Ci ratio—the ratio of the rate of assimilation and the concentration of CO_2_ in the intercellular spaces of the leaf. Different small letters indicate the statistical significance of differences evaluated by Duncan’s posthoc test (*p* < 0.05).

In this study, we addressed two major issues with respect to an episode of acute heat stress: (a) the differential effect of wheat genotypes according to their sensitivity to heat and (b) the effects of high temperature pre-acclimation. Based on the results of gas exchange measurements (Figure 1), it was clear that pre-acclimation influenced the photosynthetic capacity negatively only in the genotype Thesee, whereas in other two genotypes we observed a marginal and not statistically significant increase in net CO_2_ assimilation A (Figure 1A).

**Figure 2:**
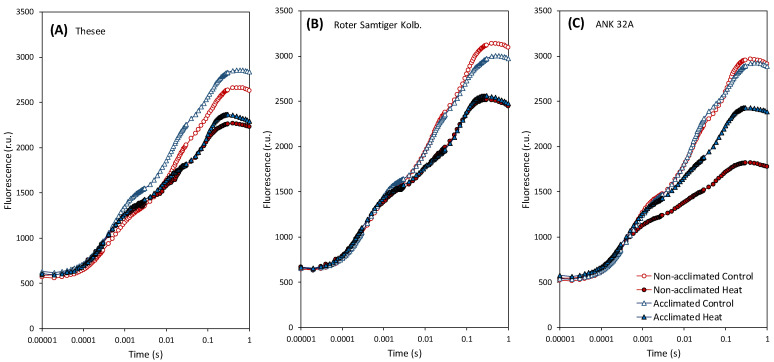
The effect of heat on chlorophyll fluorescence and comparison of individual variants and treatments in three genotypes (**A**) Thesee, (**B**) Roter Samtiger Kolbenweizen, (**C**) ANK 32A using OJIP curves. The measured fluorescence values are plotted on the logarithmic time scale.

The invariable g_s_ excludes that the decreased photosynthesis in genotype Thesee was due to a stomatal limitation of CO_2_ diffusion into the leaf (Figure 1B). The ratio between the rate of assimilation and the concentration of CO_2_ in the intercellular spaces of the leaf (A/Ci) is used to verify whether photosynthesis reduction is due to non-stomatal causes [18]. A/Ci provides valuable information on the efficiency of the use of CO_2_ by the photosynthetic apparatus. The lower A/Ci in genotype Thesee indicates that the decreased A had to be assigned to changes in biochemical constraints to CO_2_ organication and/or mesophyll CO_2_ diffusion (Figure 1C). At the same time, the A decrease in genotype Thesee was not associated with a corresponding decrease in chlorophyll fluorescence, indicating negligible irreversible impairment of PSII activity (Figure 2), and hence, we can consider the lower photosynthesis of genotype Thesee as acclimative rather than due to a chronic damage. The downregulation of photosynthetic capacity as a genotype-specific acclimation response to long-lasting elevated temperature was previously indicated by Chovancek et al. [35] in a comparative study of six wheat species/cultivars.

After the assessment of wheat response to the pre-acclimation to a moderate and prolonged increased temperature, the focus of this study was on the effects of a short-term heat wave, which was simulated in a growth chamber as an episode of exposure to 45 °C for half a day. Although the temperature 45 °C is at the edge of physiologically relevant temperature, it is very useful for short-term screening tests [36]. Expectedly, all the genotypes responded with decreased photosynthetic carbon assimilation, but with interesting differences (Figure 1A). Upon the pre-acclimation treatment, Roter Samtiger and ANK 32A gained a higher photosynthetic capacity when exposed to the heat wave; such a gain in A did not occur in genotype Thesee. Unlike short-term drought stress, in which the A decrease is caused mostly by the stomata closure [18], our results clearly show that the stomata were not closed, but slightly more open (Figure 1B). Previous studies have shown a range of reactions of stomata to elevated temperatures, including their opening [37], closure [38], or non-significant response [39]. In our case, the stomatal conductance of the plants did not decrease due to temperature.

Quite meaningfully, in the group of plants pre-acclimated to the high temperature, the subsequent severe acute heat stress tended to increase g_s_ compared to the control group of plants (Figure 1B). Though not statistically significant in comparison with the corresponding non-pre-acclimated controls, the trends were consistent in all genotypes and very likely biologically relevant. As the opening of stomata in high-temperature conditions may lead to more efficient cooling [40,41], our results suggest that the pre-acclimation of plants to high temperature improves, to some extent, the capacity of leaf thermoregulation. This inference, however, needs future specific research to confirm changes in the thermal behavior of leaves and ascertain if this response can help differentiate wheat accessions with respect to their resistance to heat waves.

More importantly and in agreement with expectations and evidence from preserved g_s_, the decrease in photosynthesis could be associated with non-stomatal inhibition. In the case of acute heat stress, a decreased A/Ci was previously proved to be associated with inhibition of some key biochemical processes [23,42]. The decrease in A/Ci ratio practically repeats the trend of decrease in photosynthesis. With a high degree of confidence, we can conclude that the effect of the decrease in metabolic functions dominates over the stomatal effects.

Among the biochemical functions most strongly affected by an acute heat stress, PSII structure and function are long been recognized as extremely sensitive [43]. To assess the effects of the heat stress on the PSII photochemistry, we applied the analysis of the fast fluorescence kinetics (Figure 2).

The rapid measurement of chlorophyll fluorescence, combined with the analysis of the fluorescence induction curves using the JIP-test, is among the fast and expeditious methods potentially very suitable to the needs of plant screening for higher tolerance to biotic and abiotic stresses [31]. The JIP-test quantifies the gradual flow of energy through PSII and other related electron transport phenomena using data from the fluorescence induction curve [44]; for recent review, [45]. In a dark-acclimated leaf, the sudden illumination by a high-intensity pulse of photosynthetically active radiation allows recording the polyphasic increase in chlorophyll fluorescence emission, which includes rising phases between steps O, J, I, P. Chlorophyll fluorescence at level O shows the minimum intensity. In this state, all electron acceptor Q_A_ molecules are oxidized. Level P represents the state in which all Q_A_ molecules are instead reduced, and fluorescence reaches its maximum intensity. Reduction of Q_A_ to Q_A_^−^ causes an increase from step O to step J and is associated with primary PSII photochemical reactions. The rapidly and slowly decreasing plastoquinone centers are indicated by the J-I and I-P rising phases, respectively [46].

**Figure 3:**
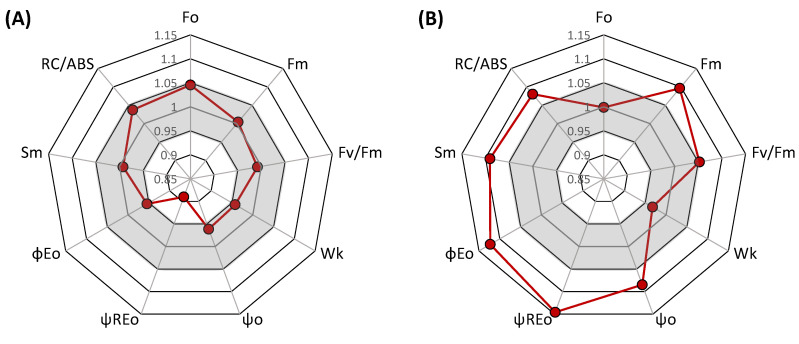
Spider plots of JIP-test parameters deduced from chlorophyll a fluorescence OJIP transient curves before the heat stress treatment (**A**) and during the heat stress (**B**) in comparison between heat pre-acclimated and non-acclimated wheat plants. For each parameter, the value of the pre-acclimated (closed circle) is set as 1 and, hence, the red points represent relative values of acclimated plants compared to non-acclimated. The gray area marks a variation of ±5%.

From the OJIP curves, it was evident that the heat stress led to a decrease in the amplitude of the fluorescence signal. In ANK 32A genotype, there was a noticeably stronger sensitivity of non-acclimated plants and, conversely, a significant increase in temperature-induced thermostability. In the other two genotypes, the differentiation of the heatwave effect between pre-acclimated and control plants was not obvious. In general, changes in the curve outline were insufficient to directly assess the processes specifically affected by the heat stress; therefore, an analysis using specific JIP-test-derived fluorescence parameters was applied (Figure 3).

**Figure 4:**
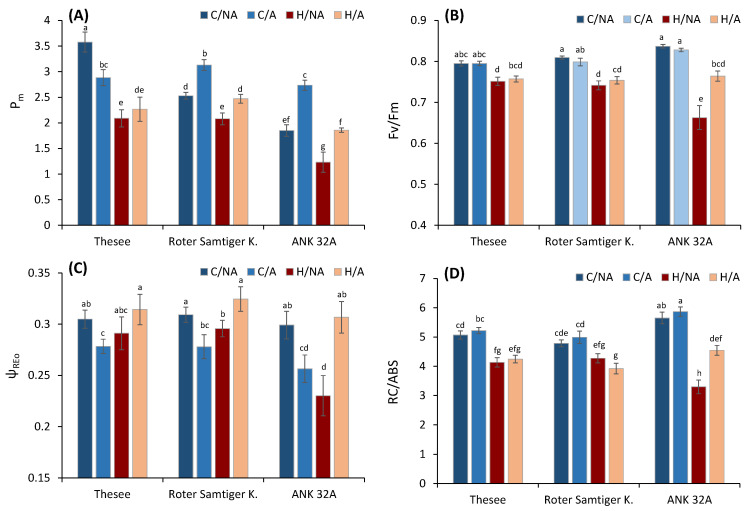
The effect of pre-acclimation and the heat stress treatment on selected biophysical parameters calculated using the analysis of P700 kinetics and chlorophyll fluorescence: (**A**) maximum amplitude of P700 signal as an indicator of the content of active PSI units; (**B**) Fv/Fm- maximum quantum yield of PSII; (**C**) ψ_REo_—efficiency/probability of electron transfer up to PSI; (**D**) RC/ABS—number of active reaction centers per absorbed light unit. C—control; H—heat stress; A—acclimated; NA—non-acclimated. Different small letters indicate the statistical significance of differences evaluated by Duncan’s posthoc test (*p* < 0.05).

The comparison using multiple parameters showed a significant effect in several of them. To assess the overall effect, we also analyzed the integrative parameter performance index (PItot), which is a recent modification of the more frequently used performance index PI_abs_. PI_tot_ is an index derived from PI_abs_ by including the efficiency of reduction of photosystem I end acceptors [47] and is considered to be more representative [48] and less sensitive to actual measuring conditions compared to the previous performance index [49]. Our results indicate apparent heat stress effects. The effects at the level of OEC shown by the parameter W_k_ was quite low (although evident), whereas the effects on the number of active reaction centers (RC/ABS) and especially the efficiency of electron transport between two photosystems (ψ_REo_) were significant. Therefore, we selected these two parameters, together with Fv/Fm being the standard indicator and Pm as an indicator of the damage of PSI reaction centers, to test the effects of interaction between pre-acclimation to high temperature and heat thermostability of photochemical processes in leaves of individual wheat genotypes (Figure 4). The statistical significance of the main factors and interactions for the selected group of parameters was also analyzed (Table 1) with interesting results for interaction of pre-acclimation and stress, and unequal trends for individual fluorescence parameters, which is also evident in Figure 4.

In all genotypes, the acute heat treatment led to a decrease in the maximum quantum yield of PSII photochemistry as Fv/Fm, which may have various causes, including structural changes in the PSII supercomplex preventing the transfer of energy from the light-harvesting complex to the PSII reaction center [50], separation of peripheral antennae from PSII reaction centers [51], inactivation of the oxygen-evolving complex [43], sustained photoprotective non-photochemical quenching [52], or inhibition of the photosynthetic electron transport chain [53]. Specific analysis of OJIP transients enabled us to recognize a decrease in the number of active reaction centers (RC/ABS) [54]. To assess the efficiency of electron transport from PSII to PSI and beyond, the parameter ψ_REo_ based on I-P amplitude was analysed. This parameter responds sensitively to various stresses [55,56].

Specifically, the parameter previously allowed for recognition of genotype-specific responses to combined stress of heat and drought [57]. From the results, the first evidence regarded the systematically lower ψ_REo_ before the exposure to the acute stress in the pre-acclimated plants of all genotypes compared to controls. This response was suggestive of acclimative adjustments in the intersystem electron transport chain. In the control (non-acclimated samples), the acute heat stress similarly caused a decrease in ψ_Reo_, which was particularly strong in ANK 32A.

Very contrasting was the response of the pre-acclimated plants of all genotypes to acute heat stress. In none of these samples exposed to the heat stress episode, we could observe any significant ψ_REo_ decrease, but instead an increase. This response suggested that the electron transport through PSI had become less sensitive to heat than PSII in the pre-acclimated groups. To strengthen this inference, we compared the concentration of photoactive PSI in leaves. The values of Pm indicated significant inactivation of PSI as a consequence of the acute stress, but it was less evident in pre-acclimated plants compared to non acclimated. The positive effect of pre-acclimation on PSI stability was evident mostly in ANK 32A.

The photosynthetic physiology of ANK 32A genotype, a yellow-green mutant, has been quite well characterized. Previous studies indicated that the ANK 32A genotype, despite its lower chlorophyll content, keeps a high photosynthetic performance in optimum conditions, though not at the level of normally green wild-type bread wheat [58]. However, upon a prolonged moderate heat stress, ANK 32A showed a sensitive response associated with a limited capacity to enhance the photoprotection, which is needed to preserve the electron transport chain during adverse conditions [59]. The same response was confirmed in this study in non-preacclimated ANK 32A plants, which were more sensitive than the next two genotypes, showing a strong decay in A, Fv/Fm, RC/ABS, PI_tot_ as consequence of the heatwave. On the other hand, the long-term pre-acclimation to elevated temperature induced an increased thermal resistance of ANK 32A plants, making the heat stress response of this genotype comparable to that of the other varieties, while strongly contrasting with the response of the non-acclimated ANK 32A. This may be associated with a higher PSI activity and content, as indicated by the parallel increase in ψ_REo_ and Pm at high-temperature conditions. Therefore, despite its higher stress sensitivity in the short term, ANK 32A has the potential to long-term acclimate efficiently to the same type of stress. For example, ANK 32A suffers from sudden increases in irradiance, but acclimates effectively to a fluctuating light regime [60]. Likewise, here we show that it suffers from acute heat stress, but acclimates effectively to a chronic exposure, revealing the importance of preserving/enhancing the PSI activity. It was previously shown that a sufficient number of active PSI reaction centers is needed to run out an efficient cyclic electron flow around PSI, which protects the electron transport chain against over-reduction [61]. Avoiding over-reduced states is one of the critical mechanisms of protection against heat stress effects [62]. As a result, a higher number of active PSII reaction centers (RC/ABS) and overall photosynthetic performance (PI_tot_) were allowed in heat-acclimated plants (Figure 4). This supports the role of PSI in photoprotection under heat stress conditions.

It must be pointed out that the methods applied in our study have not provided a full answer as to why the genotypes responded differently. There are many possible mechanisms behind it, including enhanced thylakoid membrane stability [63], efficient adjustment of the electron transport chain [64], or increase of stability of membrane proteins [65] and some others. Nevertheless, the presented study opens scope for the next experiments employing advanced biophysical and biochemical analyses to uncover the structural and molecular background of the genotypic differences in heat tolerance.

## 4. Conclusions

Short-term high temperature treatment in three wheat genotypes led to a significant non-stomatal limitation of photosynthetic carbon assimilation, but the relatively minor decrease in the parameters related to PSII photochemistry indicated mostly reversible temperature effects on photosynthesis. A comparison of the genotypes identified significantly higher heat sensitivity in ANK 32A genotype, which confirmed our previous evidence. However, the difference among the genotypes disappeared in plants previously exposed to long-term acclimation to elevated temperature. The enhanced thermal resistance, found in all genotypes but extremely evident in ANK 32A, was associated with a significant increase in the parameters related to PSI activity; this observation supports the hypothesis that an enhanced PSI activity, especially the cyclic electron transport, might play a crucial role in protecting the chloroplast membrane structure against the adverse effects of acute heat stress, such as a heatwave. Moreover, our results demonstrate that the rapid fast chlorophyll fluorescence measurements and the synthetic parameters derived in the frame of the JIP-test are useful to detect differences in sensitivity of wheat genotypes and in the capacity to mobilize the photoprotective mechanisms under stress.

## Figures and Tables

**Table 1:** The results of ANOVA analysis for the main factors and interaction between pre-acclimation and stress effects.

Factor/Interaction	A_CO2_		Fv/Fm		RC/ABS		ψ_REo_		PI_tot_	
	*p*	F	*p*	F	*p*	F	*p*	F	*p*	F
Genotype	0.007 **	5.27	0.954 ^ns^	0.05	0.025 *	3.75	0.021 *	3.92	0.037 *	3.32
Pre-acclimation	0.170 ^ns^	1.92	0.056 ^ns^	2.44	0.012 *	6.40	0.602 ^ns^	0.27	0.879 ^ns^	0.02
Stress	0.000 **	333.9	0.000 **	83.83	0.000 **	131.3	0.498 ^ns^	0.46	0.000 **	51.65
Pre-acclimation x Stress	0.025 *	5.25	0.003 **	8.72	0.494 ^ns^	0.47	0.000 **	17.93	0.000 **	17.78

^ns^ Non-significant (*p* > 0.05), *: Significant at *p* < 0.05, **: Significant at *p* < 0.01.

## Data Availability

Raw data that support the findings of this study available on request.

## Supplementary Materials

The following supporting information can be downloaded at: https://www.mdpi.com/article/10.3390/plants11050616/s1, Figure S1: The phenotype of three genotypes of winter wheat (*Triticum* sp.) used in the study in growth stage of anthesis, with fully developed flag leaves.
