# Supplementary material for: Pre-Acclimation to Elevated Temperature Stabilizes the Activity of Photosystem I in Wheat Plants Exposed to an Episode of Severe Heat Stress
Source: Plants (Basel). 2022 Feb 24;11(5):616. doi: 10.3390/plants11050616 (PMC8912596; doi:10.3390/plants11050616)
Supplement: Supplementary file 1 [file plants-11-00616-s001.zip › plants-1593166-supplementary.pdf]

Thesee | Roter Samtiger Kolbenweizen | ANK 32A

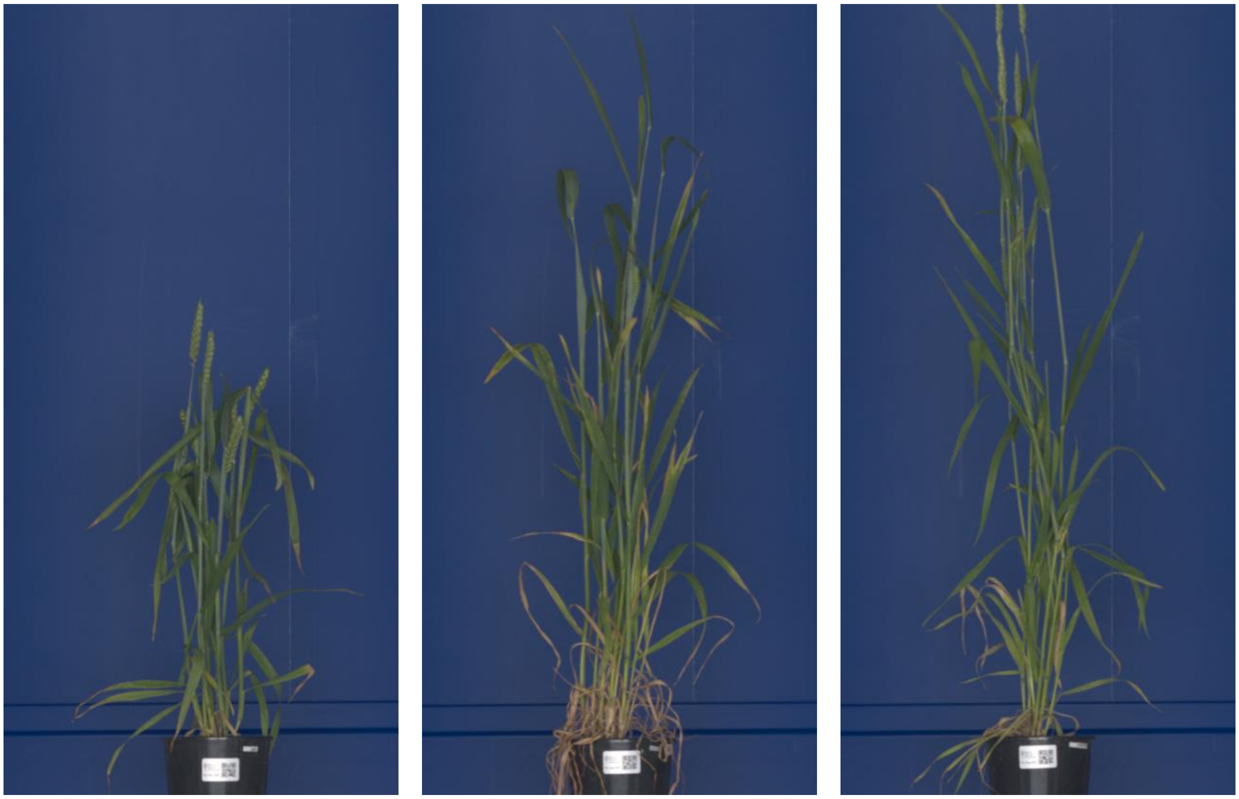

Supplementary Figure S1. The phenotype of three genotypes of winter wheat (*Triticum* sp.) used in the study in growth stage of anthesis, with fully developed flag leaves: Thesee (*Triticum aestivum* L., Germany), Roter Samtiger Kolbenweizen (*Triticum compactum* Host., Germany) and ANK 32A (*Triticum aestivum* L., Russia).
